# Selenium Deficiency Is Associated with Mortality Risk from COVID-19

**DOI:** 10.3390/nu12072098

**Published:** 2020-07-16

**Authors:** Arash Moghaddam, Raban Arved Heller, Qian Sun, Julian Seelig, Asan Cherkezov, Linda Seibert, Julian Hackler, Petra Seemann, Joachim Diegmann, Maximilian Pilz, Manuel Bachmann, Waldemar B. Minich, Lutz Schomburg

**Affiliations:** 1 ATORG, Aschaffenburg Trauma and Orthopedic Research Group, Center for Orthopedics, Trauma Surgery and Sports Medicine, Hospital Aschaffenburg-Alzenau, D-63739 Aschaffenburg, Germany; arash.moghaddam-alvandi@klinikum-ab-alz.de (A.M.); asan.cherkezov@stud-mail.uni-wuerzburg.de (A.C.); linda-glaab@t-online.de (L.S.); Joachim.Diegmann@klinikum-ab-alz.de (J.D.); manuel.bachmann.md@gmail.com (M.B.); 2 HTRG, Heidelberg Trauma Research Group, Center for Orthopedics, Trauma Surgery and Spinal Cord Injury, Heidelberg University Hospital, D-69118 Heidelberg, Germany; raban.heller@charite.de; 3 Institute for Experimental Endocrinology, Charité-Universitätsmedizin Berlin, Corporate Member of Freie Universität Berlin, Humboldt-Universität zu Berlin, and Berlin Institute of Health, D-13353 Berlin, Germany; qian.sun@charite.de (Q.S.); julian.seelig@charite.de (J.S.); julian.hackler@charite.de (J.H.); petra.seemann@charite.de (P.S.); waldemar.minich@charite.de (W.B.M.); 4 Institute of Medical Biometry and Informatics, Heidelberg University Hospital, Im Neuenheimer Feld 130.3, D-69120 Heidelberg, Germany; pilz@imbi.uni-heidelberg.de

**Keywords:** trace element, inflammation, selenoprotein P, micronutrient, COVID-19

## Abstract

SARS-CoV-2 infections underlie the current coronavirus disease (COVID-19) pandemic and are causative for a high death toll particularly among elderly subjects and those with comorbidities. Selenium (Se) is an essential trace element of high importance for human health and particularly for a well-balanced immune response. The mortality risk from a severe disease like sepsis or polytrauma is inversely related to Se status. We hypothesized that this relation also applies to COVID-19. Serum samples (*n* = 166) from COVID-19 patients (*n* = 33) were collected consecutively and analyzed for total Se by X-ray fluorescence and selenoprotein P (SELENOP) by a validated ELISA. Both biomarkers showed the expected strong correlation (*r* = 0.7758, *p* < 0.001), pointing to an insufficient Se availability for optimal selenoprotein expression. In comparison with reference data from a European cross-sectional analysis (EPIC, *n* = 1915), the patients showed a pronounced deficit in total serum Se (mean ± SD, 50.8 ± 15.7 vs. 84.4 ± 23.4 µg/L) and SELENOP (3.0 ± 1.4 vs. 4.3 ± 1.0 mg/L) concentrations. A Se status below the 2.5th percentile of the reference population, i.e., [Se] < 45.7 µg/L and [SELENOP] < 2.56 mg/L, was present in 43.4% and 39.2% of COVID samples, respectively. The Se status was significantly higher in samples from surviving COVID patients as compared with non-survivors (Se; 53.3 ± 16.2 vs. 40.8 ± 8.1 µg/L, SELENOP; 3.3 ± 1.3 vs. 2.1 ± 0.9 mg/L), recovering with time in survivors while remaining low or even declining in non-survivors. We conclude that Se status analysis in COVID patients provides diagnostic information. However, causality remains unknown due to the observational nature of this study. Nevertheless, the findings strengthen the notion of a relevant role of Se for COVID convalescence and support the discussion on adjuvant Se supplementation in severely diseased and Se-deficient patients.

## 1. Introduction

Severe acute respiratory syndrome coronavirus-2 (SARS-CoV-2) infections underlie the current coronavirus disease (COVID-19) pandemic and are causative for an increasingly high death toll particularly among elderly subjects and those who have severe comorbidities, e.g., chronic obstructive pulmonary disease, hypertension, diabetes, cancer, or a combination thereof [1,2]. It has been reported that severe disease course often associates with an overreaction of the body’s immune system with a massive cytokine and chemokine release (“cytokine storm”) [3]. Accordingly, the attempts at controlling the inflammation by immunosuppressive treatment using, e.g., high dosages of corticosteroids have shown promising effects in reducing the rate of fatal disease course among the severely diseased COVID patients on mechanical ventilation (medRxiv 2020.06.22.20137273), causing a surge in dexamethasone demand [4]. This treatment success is reminiscent of the positive reports on dexamethasone being capable of positively affecting the course of severe acute respiratory distress syndrome [5], or of reducing the mortality rate in severely diseased and delirious patients from typhoid fever [6]. The strategy of repurposing common drugs known to positively affect the immune response are now increasingly applied in the current COVID pandemic [7]. The positive effects with tocilizumab and sarilumab are the most recent examples (NCT04306705, NCT04322773). An adjuvant supply of certain micronutrients as positive modulators of the immune system may further support these attempts, and some vitamins (A, B6, B12, C, D, and E) and essential trace elements (zinc, iron, selenium (Se), magnesium, or copper) are discussed as particularly promising [8]. However, at present, the data base is very small in relation to these micronutrients, and it is unknown whether certain vitamins or trace elements are indeed deficient in patients with COVID-19, and whether the concentrations are related to disease severity or mortality risk.

For several reasons, the essential trace element Se is of particular relevance for viral infections among these nutritional factors. The immune system relies on a set of specific selenoproteins containing selenocysteine in their active sites and known to depend on abundant Se supply for their full expression and enzymatic activities [9,10]. Se deficiency is an established risk factor for viral infections [11]. Pathogens show higher mutation rates in Se-deficient subjects and can decisively contribute to a rapid evolution of pathogenic viral species [12]. Keshan disease is an endemic cardiomyopathy related to Se deficiency, and supplemental Se has proven meaningful for reducing the virus-associated disease incidence [13]. Se deficiency is also a risk factor for death from severe disease, as shown, e.g., in sepsis [14] or polytraumatic injury [15]. Notably, the cure rate from COVID-19 was recently associated with basal Se status in different areas of China [16]. Collectively, the available studies support the notion that Se may be of relevance for infection with SARS-CoV-2 and disease course of COVID-19 [17,18,19]. However, data on Se status of individual patients severely affected by COVID-19 are missing. We hypothesized that severe Se deficiency is prevalent among the patients and associates with poor survival odds in COVID-19.

## 2. Materials and Methods

### 2.1. Study Design

A cross-sectional study of patients with COVID-19 was conducted at the non-profit Public Hospital Klinikum Aschaffenburg-Alzenau, Germany. Diagnosis of COVID-19 was based on positive detection of viral RNA using RT-PCR (real-time PCR—E-Gen according to Corman et al. [20], Medizinisches Versorgungszentrum MVZ Labor PD Dr. Volkmann & Kollegen GbR, Karlsruhe, Germany). The study was conducted in accordance with the Declaration of Helsinki. Ethical counselling was provided by the authorities in Bavaria, Germany (Ethik-Kommission der Bayerischen Landesärztekammer, EA No. #20033), and the study was registered at the German Clinical Trial Register (Deutsches Register Klinischer Studien, ID: DRKS00022294). All patients enrolled into the analysis or next of kin provided written informed consent. The number of blood drawings per patient was [median (IQR)] 4 (4) or [mean ± SD] 5.03 ± 4.27 samples/patient. The samples were stored at −80 °C (Aschaffenburg, Germany) and sent on dry ice to a remote lab from the clinics for analysis (Charité Universitätsmedizin Berlin, Berlin, Germany). All measurements were conducted by scientists and technicians blinded to the clinical information. Reference values were derived from a comprehensive dataset of adult subjects participating in the European Prospective Investigation into Cancer and Nutrition (EPIC) study, analyzed by the same technology as published recently [21].

### 2.2. Trace Element Analysis

Total reflection X-ray fluorescence (TXRF) was used to determine the concentration of Se in serum samples using a benchtop TXRF spectrometer (S4 T-STAR, Bruker Nano GmbH, Berlin, Germany). Briefly, samples were diluted with a gallium standard, applied to polished quartz glass slides and dried overnight. Seronorm serum standard (Sero AS, Billingstad, Norway) served as control. The concentrations measured were within the specified range of the standard, and the inter-assay coefficient of variation (CV) was below 5% at a concentration of 45 µg Se/L serum.

### 2.3. SELENOP Quantification by ELISA

SELENOP concentrations were measured from the serum samples by a sandwich method with monoclonal antibodies against human SELENOP using a validated commercial SELENOP-specific ELISA (selenOtest ELISA, selenOmed GmbH, Berlin, Germany) as described [22]. Quality of measurements was verified by including two human serum standards in each assay run. The inter-assay CV was below 15% during the analyses.

### 2.4. Assessment of Glutathione Peroxidase-3 (GPx3) Activity

The activity of glutathione peroxidase-3 (GPx3) was assessed by a coupled enzymatic test procedure monitoring nicotinamide adenine dinucleotide phosphate (NADPH) consumption at 340 nm, as described earlier [23,24]. Briefly, serum samples were incubated with enzyme buffer containing 3.4 mM reduced glutathione (GSH), 0.27 mg/mL NADPH, 1 mM NaN_3_, and 0.3 U/mL glutathione reductase. The enzymatic reaction was started by hydrogen peroxide, and consumption of NADPH was monitored at 340 nm. Inter- and intra-assay CVs were below 20%.

### 2.5. Statistical Analysis

Statistical analysis was performed with GraphPad Prism (Version 7, GraphPad Software Inc., San Diego, CA, USA) and the open software R, version 3.6.0 [25], applying the packages “tidyr” [26], “dplyr” [27], “pROC” [28], and “ggplot2” [29]. The Shapiro–Wilk test was used for assessing the normal distribution of values. Categorical variables were evaluated by Boschloo’s test [30]. Comparisons were conducted by unpaired Student’s t-test. More than two groups were compared with ANOVA and Dunn’s multiple comparisons test. Correlations were tested by Spearman’s correlation test. Differences between ROC curves were assessed by applying the DeLong’s test for two correlated ROC curves. All statistical tests were two-sided, and *p*-values < 0.05 were considered significant; * *p* < 0.05, ** *p* < 0.01, *** *p* < 0.001, and **** *p* < 0.0001.

## 3. Results

### 3.1. Patient Characteristics

A total of *n* = 33 patients qualified for analysis and were enrolled into this observational study, providing a set of *n* = 166 consecutive serum samples. COVID-19 patients who survived or died showed similar characteristics, except for a lower age range of the survivors (Table 1).

### 3.2. Selenium (Se) Status Analysis

**Figure 1:**
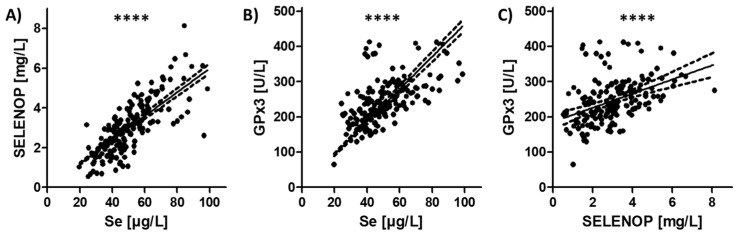
Analysis of Se status from samples of patients suffering from COVID-19 by three complementary serum biomarkers. Serum samples (*n* = 166) were analyzed from COVID-19 patients (*n* = 33) by measuring total Se concentration, serum SELENOP level, and activity of secreted GPx3. (**A**) The Se transporter SELENOP and total Se concentration showed a tight positive linear correlation (*r* = 0.7896), in agreement with the analysis of (**B**) GPx3 activity and total Se concentration (*r* = 0.6239), as well as with (**C**) GPx3 activity and SELENOP concentration (*r* = 0.4954). *r*: Spearman correlation coefficient (2-sided, 2-tailed), **** *p* < 0.0001.

Serum Se status was evaluated from all patient samples as assessed by three complementary biomarkers, i.e., total serum Se and SELENOP concentrations, as well as GPx3 activity. The three Se status biomarkers showed significant and linear correlations over the full range of data, indicating a high quality of the samples (Figure 1). The correlation coefficients were highest for the parameter pair of total serum Se and SELENOP concentration (Figure 1A), followed by the parameter pair GPx3 activity and total serum Se (Figure 1B). GPx3 activity and serum SELENOP concentration showed the least stringent correlation (Figure 1C).

### 3.3. Se Status of COVID-19 Patients in Relation to Reference Range of Healthy Control Subjects

An average population-wide Se status was deduced from *n* = 1915 datasets obtained earlier from healthy adult subjects participating in the cross-sectional EPIC study [21]. Reference ranges for total serum Se and SELENOP concentrations were deduced by determination of the 2.5th–97.5th percentile of the data. According to this large cross-sectional study, SELENOP concentrations are unrelated to age [21]. The chosen criterion of 95% of data constituting the reference ranges classifies a normal Se status when residing in the range of 45.7–131.6 µg/L for serum Se, and 2.56–6.63 mg/L for serum SELENOP concentration, respectively. According to these reference ranges, 44.4% of samples from COVID-19 patients were deficient in Se, and 39.6% were deficient in SELENOP.

### 3.4. Se Status of COVID-19 Patients in Relation to Survival

When separating patient samples from surviving vs. deceased COVID-19 patients, the difference becomes more obvious. In the samples of deceased COVID-19 patients, 64.7% and 70.6% showed Se and SELENOP deficiency, respectively, whereas 39.3% and 32.6% of the samples from the survivors had to be classified as Se- and SELENOP-deficient, respectively. Accordingly, a significantly lower Se status was identified in the non-survivors in comparison with the survivors with respect to all three biomarkers of Se status analyzed (Table 2).

**Figure 2:**
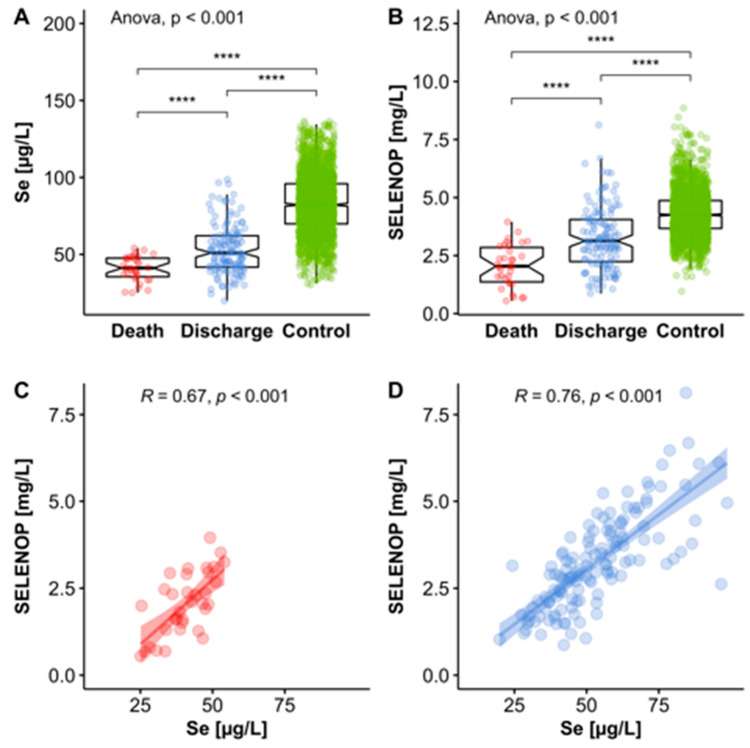
Comparison of Se status in COVID-19 patients who survived or died in relation to healthy controls. (**A**) Total serum Se concentrations differed significantly and were most strongly depressed in COVID-19 patients who did not survive. (**B**) SELENOP concentrations differed to a similar extent and were also lowest in non-survivors. (**C**) As observed in the full cohort of samples, Se and SELENOP showed a strong positive correlation in the group of non-survivors, as well as (**D**) in the group of survivors, albeit across a smaller and more limited concentration range in the non-survivors. All tests were two-sided and *p*-values < 0.05 were considered statistically significant; *R*: Spearman correlation coefficient (2-sided, 2-tailed), **** indicates *p* < 0.0001.

A comparison of the median values and inter quartile ranges (IQR) of the samples from the COVID-19 patients who did not survive in relation to the reference cohort of healthy adult European subjects indicates that the groups differ strongly, i.e., the IQR do not overlap. This means that the ranges encompassing 75% of all samples are separated from each other, irrespective of the biomarker used, i.e., both in relation to total serum Se and serum SELENOP concentrations (Figure 2A,B). Notably, the bottom 75% of values from the deceased patients are below the median values of the surviving COVID-19 patients, suggesting that both parameters of Se status are of value for the identification of patients with severe disease course and high mortality risk.

With regard to the choice of biomarker, both total serum Se and SELENOP concentrations appear similarly suitable for providing information on survival chances of COVID-19 patients. Importantly, Se and SELENOP showed the known positive linear correlation in both the group of non-survivors and of the surviving patients that were successfully discharged (Figure 2C,D).

**Figure 3:**
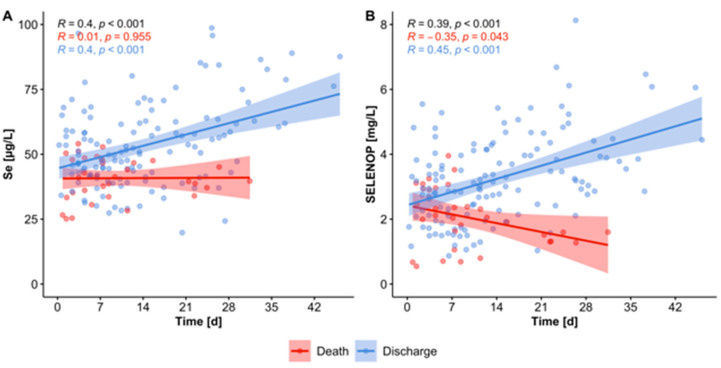
Time-resolved changes in Se status in surviving vs. deceased patients. Serum samples from COVID-19 patients were analyzed for (**A**) total Se, and (**B**) serum SELENOP concentrations. Surviving patients (blue dots) showed increasing Se status with time, with respect to both serum Se and SELENOP. In comparison, Se status remained constant, and SELENOP concentrations declined, respectively, in non-surviving patients (red dots). All tests were two-sided and *p*-values < 0.05 were considered statistically significant; R: Spearman correlation coefficient (2-sided, 2-tailed); overall, death- and discharge-related correlations of Se status vs. time are indicated in the upper left corners.

The samples available for analysis were from different points in time as leftover serum from routine laboratory analyses. Hereby, it was possible to conduct a time-resolved analysis of changes in Se status of surviving vs. deceased COVID-19 patients. The analysis highlights that the Se status in patients surviving the disease tended to recover from the low values observed at admittance to the hospital, whereas no such positive development was observed in the non-survivors (Figure 3).

A direct comparison of Se status in COVID-19 patients to reference values for the activity of GPx3 as a biomarker was not possible, as GPx3 had not been determined in the samples of the large reference cohort from the EPIC study [21].

**Figure 4:**
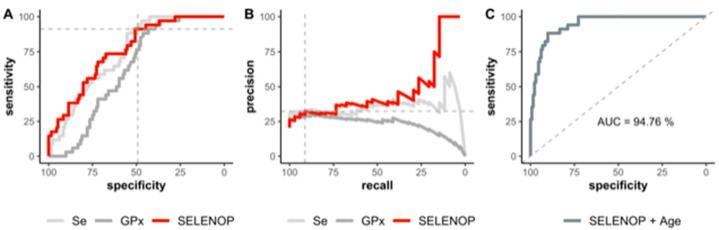
Receiver operating characteristics (ROC) analyses of Se status biomarkers in relation to risk of death from COVID-19. (**A**) ROC analyses as univariate prediction models based on the serum concentrations of SELENOP, Se, and GPx3 (pooled values from admission to the endpoint of the study) are capable of discriminating between patients that died and those that have been discharged. The optimal cutpoint of SELENOP concentrations at 3.1 mg/L according to Youden’s J statistics is indicated by the point where the dashed grey lines cross. (**B**) The corresponding precision recall curve (PRC) indicates the fraction of true positives among all the positive predictions and may serve as a meaningful addition to current risk estimates. The corresponding cutpoint is again indicated. (**C**) ROC analysis of SELENOP status in relation to risk of death from COVID-19 with respect to the patients’ age. The area under the curve (AUC) is indicated below the diagonal 50% line.

Next, a receiver operating characteristic (ROC) curve analysis was conducted to analyze the diagnostic ability of the Se status biomarkers for survival odds. ROC curve analyses can contribute to decision-making in a binary classifier system by testing a discrimination threshold via calculating all possible variations. However, ROC plots alone may be misleading and bear the risk of error when applied in imbalanced classification scenarios [31]. For this reason, a precision recall curve (PRC) was calculated to identify the fraction of true positives among all the positive predictions, thereby providing a more accurate prediction of future classification performance (Figure 4). The available data on SELENOP, Se, and GPx3 were suitable to reliably distinguish between those patients who could be discharged and those who died, respectively. Applying a stepwise Akaike information criteria (AIC) selection process revealed that the SELENOP concentration outperformed the other variables as well as combinations thereof. This result is mirrored in both the corresponding ROC and PRC curves (Figure 4A,B). Calculating the area under the curve (AUC) for the three Se status biomarkers indicates a better suitability of total serum Se and SELENOP concentrations in comparison with GPx3 activity for diagnosis and prediction, i.e., 75.9% for SELENOP and 74.2% for total Se, respectively, vs. 62.4% for GPx3 activity.

As it is known that age strongly predisposes to severe disease course and mortality risk in COVID-19, an analysis for serum Se and SELENOP in combination with age was conducted. The model based on SELENOP was slightly superior to the model based on Se. Significant differences were detected between the models based on either SELENOP or Se and GPx3 (DeLong’s test; AUC (SELENOP) = 75.9% vs. AUC (GPx3) = 62.4%, *p* = 0.004; AUC (Se) = 74.2% vs. AUC (GPx3) = 62.4%, *p* = 0.007). The final univariate model based on SELENOP yielded an AUC of 75.9%. The optimal cutpoint based on Youden’s J statistic was chosen at 3.1 mg/L (Figure 4A). This cutpoint is characterized by a sensitivity of 91.2% and a specificity of 50.8%, and may serve as a valuable screening tool to contribute to a better assessment of the mortality risk in patients suffering from COVID-19. This is also reflected in the precision recall curve (PRC) recommended in such analyses [31] (Figure 4B). In view of the limited sample size of *n* = 33 patients only, the Se status biomarkers were analyzed in a univariate modeling process via stepwise backwards AIC selection to avoid an overfit. The favored SELENOP-based model was finally adjusted for the patients’ age and resulted in an increased AUC of 94.8% (Figure 4C).

This notion is further underlined by the specific characteristics of the predictive models used (Table 3).

## 4. Discussion

In this manuscript, we report that patients suffering from COVID-19 display a deficiency in the essential trace element Se in blood, along with low concentrations of the Se transporter SELENOP and low enzymatic activity of the secreted GPx3. Notably, the Se deficiency was very strong in comparison with healthy European adults, and it was reflected concordantly in relatively depressed readings of all three different Se status biomarkers determined. The observation that Se deficiency was more severe in the samples obtained from non-survivors as compared with survivors of COVID-19 may suggest some relevance of the trace element for coping with the virus and for successful convalescence. This hypothesis is also supported by the difference in Se status development in time, with survivors displaying a progressively recovering Se status, while the non-survivors do not.

Besides the physiological role of Se for supporting the biosynthesis of immune system-relevant selenoproteins, the data also highlight that a determination of Se status by any of the biomarkers evaluated is of diagnostic value for a better prediction of disease course and an improved identification of patients at particular risk for losing the battle against this devastating infection. However, reliable Se analysis is not readily available at all clinics and hospitals, and many commercial or experimental analytical test systems for SELENOP quantification are not yielding accurate results [32], causing confusion and disarray [33]. For this reason, the issue of avoiding severe Se deficiency in the preventive and clinical settings by using a respectively balanced diet or suitable supplements may be the most urgent and meaningful consequence from the interaction between Se deficiency and mortality risk observed in this study.

Although the nature of the analysis as an observational study does not allow the deduction of causal relationships, there are different hypotheses for the underlying biochemical pathways leading to the observations presented in this manuscript.

Firstly, Se status may already have been relatively low in the patients before disease, constituting a risk factor for viral infection as shown previously for other diseases [11,12]. In this respect, the experiences with viral-induced Keshan disease [13] or AIDS [34] may serve as paradigmatic examples highlighting the potential relevance of Se for infection risk and disease course [34]. However, the high infection rate of SARS-CoV-2 apparently infecting very many of the directly exposed subjects [35] in combination with the majority of COVID-19 samples exhibiting Se values below the 2.5th percentile of the population range argues against a Se-dependent predisposition as an explanation for the findings.

Secondly, in disease and upon the growing inflammation, a potentially pre-existing low Se status may decline further. This notion is supported from similar findings in other severe diseases, especially sepsis [14] and polytraumatic injury [15], where low, declining, and mortality-relevant Se deficiency has been observed that is unlikely a predisposition. Moreover, the negative acute phase response of hepatic SELENOP biosynthesis [36], together with the suppressive effects of hypoxia [37] or cytokines, e.g., IL-6 [38], argue in favor of this mechanism contributing to the differences.

Thirdly, a longer stay on the ICU under inflammatory and hypoxic conditions may cause an elevated Se requirement due to ongoing Se loss, as erythrocyte Se often remains normal despite declining Se in blood [39]. In human evolution, high-quality medical care with supportive ventilation was usually not available, and an infection was followed soon by either remission or death. Under these conditions, safeguarding essential micronutrients for later recovery was no survival advantage. The present care on the ICU over long periods of time constitutes a fundamental different situation, where the constant suppression of hepatic SELENOP biosynthesis may require supplemental measures in the long run [39]. Concordant with this notion, the hypothesized association of low Se status with impaired recovery was reported from an in silico analysis of cure rates from COVID-19 in the different areas of China with diverging baseline Se status [16].

Fourthly, an over-shooting immune response may be directly related to Se status as oxidative stress may overrun the capacity of protective selenoenzymes of the GPx and thioredoxin reductase families and low molecular weight antioxidants [40]. This loss of redox balance has been hypothesized before as of potential etiopathogenic relevance [12,41]. The therapeutic success of dexamethasone or tocilicumab treatment, as well as the perspective of the GPx mimetic ebselen as a promising therapeutic measure lend further support to this theory [42,43].

**Figure 5:**
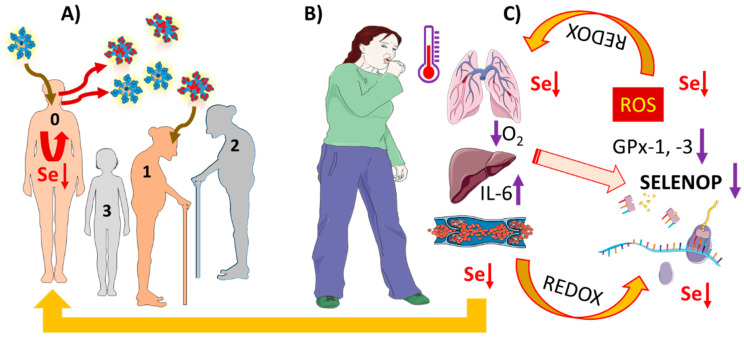
Pathophysiological mechanisms potentially underlying low Se status in severe COVID-19. Infections by SARS-CoV-2 occur largely independent from baseline Se status. (**A**) Some individuals with a poor immune system and low baseline Se status (0) may spread the virus (blue) efficiently and allow viral replication and rapid evolution of particular pathogenic viral species (red) due to low expression of protective selenoenzymes. Subjects with better Se status (1–3) may be less prone to severe disease course. (**B**) COVID-19 is characterized by inflammation, hypoxia, and high cytokine concentrations (e.g., IL-6). The combination of hypoxia and IL-6 suppresses selenoprotein expression. (**C**) Biosynthesis of the Se transporter SELENOP in hepatocytes is particularly sensitive, causing whole body Se status decline and insufficient expression of protective selenoenzymes, e.g., cytosolic GPx1 and plasma GPx3. Insufficient inactivation of peroxides as precursors of reactive oxygen species (ROS) results, causing a serious disturbance of redox balance, closing a vicious cycle both with respect to selenoprotein expression, Se concentrations, and COVID-19 progression. It is hypothesized that supplemental Se may interrupt this series of detrimental events and contribute to better odds for convalescence. This figure was created by using some Servier Medical Art templates, which are licensed under a Creative Commons Attribution 3.0 Unported License; https://smart.servier.com.

Finally, a declining serum Se status may just constitute a surrogate marker for disease severity and the tone of pathological stressors, like hypoxia and inflammatory cytokines. This notion is supported by a vast body of literature on declining selenoprotein biosynthesis under acute phase conditions, in inflammation and under hypoxia. A declining Se status will further disrupt the redox balance thereby closing a fatal feed-forward loop, again arguing for the potential relevance of some supplemental support to interrupt this vicious cycle during long-lasting disease (Figure 5).

Collectively, similar to the proposed interrelation of declining Se status in malignant diseases, the strong deficit in Se and SELENOP observed in COVID-19 may result from a combination of the aforementioned pathways and interactions. Supportive measures aimed at improving selenoprotein biosynthesis in COVID-19 may enable a better redox control and fine-tuned response of the immune system [41]. It appears meaningful, timely, and promising to initiate population-wide measures trying to identify subjects with pre-existing Se deficits, not just as a preventive measure for viral infections, spread, and virulence development [11,12,42], but also to reduce the individual risk for cardiovascular mortality [44,45,46,47], cancer [21,48,49], and death from severe disease [10,14,39].

The particular strengths of the current study are the parallel assessment of different and coherent biomarkers of Se status by a standardized methodology, and the blinded set-up of the analyses. Among the limitations are, as usual in explorative pilot studies, the relatively limited number of patients and samples, and the lack of clinical data on inflammatory parameters.

## 5. Conclusions

COVID-19 constitutes a universal threat to human health, necessitating fast, promising, and safe measures for reducing infection risk, suppressing virulence development, strengthening the immune system, and supporting recovery. The essential trace element Se may be most relevant for these issues. Subjects residing in areas with poor baseline Se supply or on restricted nutrition, and COVID patients with pre-existing comorbidities or long disease course are at particularly elevated risk for severe Se deficiency, and may profit from improving the Se supply by dietary or supplemental measures. The observed association of mortality risk with Se deficit and the likely underlying feed-forward mechanism argues for initiating intervention studies under the highest quality standards, in order not to miss a universally available, inexpensive, and safe preventive measure and adjuvant treatment option.

## Figures and Tables

**Table 1:** Characteristics of the COVID-19 patients contributing to this study.

	Death	Discharge	Total
**Sex**			
female	4 (67%)	15 (56%)	19 (58%)
male	2 (33%)	12 (44%)	14 (42%)
**Age**			
median (IQR)	89 (81, 94)	69 (38, 91)	77 (38, 94)
**Comorbidities**			
hypertension	4 (67%)	18 (67%)	22 (67%)
diabetes	2 (33%)	4 (15%)	6 (18%)
COPD	0 (0%)	1 (4%)	1 (3%)
CVD	3 (50%)	14 (52%)	17 (52%)
cerebrovascular disease	1 (17%)	5 (19%)	6 (18%)
adipositas	1 (17%)	6 (22%)	7 (21%)
Time to discharge or death * [d]			
median (IQR)	10 (2, 32)	19 (3, 46)	15 (2, 46)

* Death in combination with COVID-19 diagnosis, irrespective of final mortality cause.

**Table 2:** Comparison of Se status biomarkers in COVID-19 samples in relation to survival.

	All Samples	Discharge	Death	*p*-Value *
**serum Se [µg/L]**	*n* = 166	***n*** **= 132**	***n*** **= 34**	***p*** **< 0.001**
	50.8 ± 15.7	**53.3 ± 16.2**	**40.8 ± 8.1**	
**serum SELENOP [mg/L]**	3.0 ± 1.4	**3.3 ± 1.3**	**2.1 ± 0.9**	***p*** **< 0.001**
**serum GPx3 [U/L]**	246.1 ± 64.4	**251.6 ± 69.6**	**224.8 ± 30.3**	***p*** **< 0.001**

* Student’s *t*-test, 2-tailed, 2-sided, comparison of discharge vs. death (bold numbers).

**Table 3:** Specific characteristics of the predictive models used. For each model, the variable estimates included in the calculations are provided with their corresponding confidence interval (CI).

	Serum Se	Serum SELENOP	GPx3 Activity	All
**(intercept)**	−1.70 *** [−2.20, −1.20]	−1.75 *** [−2.27, −1.24]	−1.42 *** [−1.81, −1.02]	−1.80 *** [−2.34, −1.26]
**Se**	−1.19 *** [−1.79, −0.60]			−0.55 [−1.39, 0.30]
**SELENOP**		−1.28 *** [−1.86, −0.70]		−0.94 * [−1.72, −0.16]
**GPx3**			−0.46 * [−0.89, −0.04]	0.09 [−0.37, 0.54]
**N**	166	166	166	166
**AIC**	150.5	146.3	167.3	148.5
**Pseudo R^2^**	0.19	0.23	0.05	0.24

All continuous predictors are mean-centered and scaled by 1 standard deviation. *** *p* < 0.001; * *p* < 0.05.
